# Classic yin and yang tonic formula for osteopenia: study protocol for a randomized controlled trial

**DOI:** 10.1186/1745-6215-12-187

**Published:** 2011-08-02

**Authors:** Feng Yang, De-Zhi Tang, Xue-Jun Cui, Jonathan D Holz, Qin Bian, Qi Shi, Yong-Jun Wang

**Affiliations:** 1 Longhua Hospital, Shanghai University of Traditional Chinese Medicine, Shanghai, 200032, PR China; 2 Shaanxi University of Traditional Chinese Medicine, Xianyang, 712000, PR China; 3 Spine Research Institute, Shanghai University of Traditional Chinese Medicine, Shanghai, 200032, PR China; 4 Department of Orthopaedics, Center for Musculoskeletal Research, University of Rochester, Rochester, New York 14642, USA; 5 Department of Environmental Medicine, University of Rochester, Rochester, New York 14642, USA

## Abstract

**Background:**

Osteoporosis is a growing worldwide problem, with the greatest burden resulting from fractures. Nevertheless, the majority of fractures in adults occur in those with "osteopenia" (bone mineral density (BMD) only moderately lower than young normal individuals). Since long-term drug therapy is an expensive option with uncertain consequences and side effects, natural herbal therapy offers an attractive alternative. The purpose of this study is to evaluate the effect on BMD and safety of the Classic Yin and Yang Tonic Formula for treatment of osteopenia and to investigate the mechanism by which this efficacy is achieved.

**Methods/design:**

We propose a multicenter double-blind randomized placebo-controlled trial to evaluate the efficacy and safety of the Classic Yin and Yang Tonic Formula for the treatment of osteopenia. Participants aged 55 to 75 with low bone mineral density (T-score between -1 and -2.5) and kidney deficiency in TCM will be included and randomly allocated into two groups: treatment group and control group. Participants in the treatment group will be treated with Classic Yin and Yang Tonic Granule, while the controlled group will receive placebo. Primary outcome measure will be BMD of the lumbar spine and proximal femur using dual-energy X-ray absorptiometry. Secondary outcomes will include pain intensity measured with visual analogue scales, quality of life, serum markers of bone metabolism, indices of Neuro-endocrino-immune network and safety.

**Discussion:**

If the Classic Yin and Yang Tonic Formula can increase bone mass without adverse effects, it may be a novel strategy for the treatment of osteoporosis. Furthermore, the mechanism of the Chinese medical formula for osteoporosis will be partially elucidated.

**Trial registration:**

This study is registered at ClinicalTrials.gov, NCT01271647.

## Background

Osteoporosis is a growing problem worldwide, with the greatest burden resulting from fractures. Nevertheless, the majority of fractures in adults occur in those with "osteopenia" (bone mineral density (BMD) only moderately lower than young normal individuals). Thus, there has been an interest in developing approaches to prevent bone loss. The agents currently approved for treatment and/or prevention of osteopenia include bisphosphonates, hormone replacement therapy with estrogen or combination estrogen/progesterone preparations, calcitonin, raloxifene, and parathyroid hormone as well. But short- and long-term consequences (adverse effects) of these therapies continue to be discovered. The adverse outcomes include cancer, and cardiac, dermatologic, gastrointestinal, gynecologic, immunologic, metabolic, musculoskeletal, neurological, psychiatric, and respiratory events.

Since long-term drug therapy is an expensive option with uncertain consequences and side effects, natural herbal therapy offers a possible alternative. The Classic Yin and Yang Tonic Formula (CYYTF) have been used to treat osteoporosis or osteopenia in traditional Chinese medicine (TCM) for a long time. Several putative mechanisms for this have been proposed. These include stimulation of osteoblast proliferation and differentiation, osteogenesis, and inhibition of bone resorption, as a estrogen-like function. For this reason, Chinese guidelines for the treatment of osteopenia include natural herbal therapy. However, there is currently no randomized placebo-controlled trial to verify its efficacy in treating bone mass loss. The present study is to examine effects of a Chinese herbal intervention using the Classic Yin and Yang Tonic Formula (CYYTF) to improve bone mineral density of patients with osteopenia in a randomized, double-blind, placebo-controlled trial. Results of this study will provide evidence regarding the value of the Classic Yin and Yang Tonic Formula (CYYTF) as an intervention to increase BMD of osteopenic individuals. Furthermore, the mechanisms of action can be partially identified by this study.

## Methods and Design

### Study Design

This clinical trial is a multi-center, randomized, double-blind, placebo-controlled design. Subjects will be enrolled at four hospitals: 1) Longhua hospital affiliated with Shanghai university of TCM; 2) Huadong hospital affiliated with Fudan university; 3) The central hospital of Yangpu district, Shanghai; 4) The Red Cross hopital of Xi'an. This study is conducted in 4 sites in China.

### Ethics Issue

This study has been approved by the Ethics Board of Shanghai University of TCM (No: 2011LCSY002). Each participating centre obtained a local Institutional Review Board Approval. All study participants will sign the written informed consent prior to participation.

### Patients Population and Recruitment Procedure

The study population consists of individuals aged 55 to 75 with BMD below 80% of the young adult mean (YAM) (T score -1.0 to -2.5 at the lumbar spine or hip). Data for the YAM and T score values were obtained from reference data of healthy women 20 to 29 years of age [1]. The individuals whose syndrome differentiation are kidney deficiency will be included. In traditional Chinese medicine (TCM), kidney deficiency syndrome is a general term for deficiency conditions of kidney and Neuro-endocrino-immune network, The symptoms mainly include: low back pain, soreness and weakness of the lumbar regions and knees, dizziness, fatigue, spontaneous sweating, a hot or cold sensation in the palms, soles and chest, dysphoria, insomnia, The pulse is weak, the tongue is red and covered without fur or bulky, moist, and covered with white fur.

Subjects will be excluded if they have disorders such as primary hyperparathyroidism; Cushing's syndrome; premature menopause due to hypothalamic, pituitary, or gonadal insufficiency; poorly controlled diabetes mellitus (HbA1c>8.0%); or other causes of secondary osteoporosis. Subjects will also be excluded if they have taken bisphosphonates at any time. The trial will exclude individuals who have taken glucocorticoids, calcitonin, vitamin K, active vitamin D compounds, or hormone replacement therapy within the previous 2 months. In addition, serum calcium (Ca) levels above 10.6 mg/dL (2.7 mmol/L) or below 8.0 mg/dl (2.0 mmol/L), serum creatinine levels above1.5 mg/dL (133 μmol/L), or clinically significant hepatic disorders, are also exclusion. Furthermore, subjects that have Malignancies, or physical or mental disabilities will be excluded, as well as lactating or pregnant patients.

This study is to be conducted in accordance with protection of patients, as outlined in the Declaration of Helsinki, and approved by the appropriate Institutional Review Boards. Each participant will sign the written informed consent before undergoing any examination or study procedure, in compliance with Good Clinical Practice. We will utilize EpicCare databases to identify all persons aged 55-75 who had a DXA scan during the prior two years at any of the four hospitals, and with BMD of the hip (femoral neck or trochanter) and/or spine falling within the T-score range of -1.0 and -2.5. The patients whose syndrome differentiation of TCM is kidney deficiency are included. Patients who initially meet these eligibility criteria are then to complete the additional baseline testing and will be randomized into either the treatment or the control group.

### Interventions

Eligible patients will be randomized to one of the two arms: placebo and Chinese medical herb (18 g twice daily). All drugs will be administered orally for 6 months. In this study, the treating physicians, subjects, investigators and statisticians will be blinded to treatment assignment. In this respect, the trial is double-blind. Randomization of subjects will occur centrally using a random number generator and will be stratified by syndrome differentiation of TCM (Syndrome, as related to illnesses in Western medicine, is composed of a set of signs and/or symptoms classified by Traditional Chinese Medicine practitioners [2]. TCM Syndrome differentiation is based on symptoms and helps to identify a subset of disease). Patients visit the doctor at 6 and 12 months follow-up.

### Randomization and Allocation

**Figure 1:**
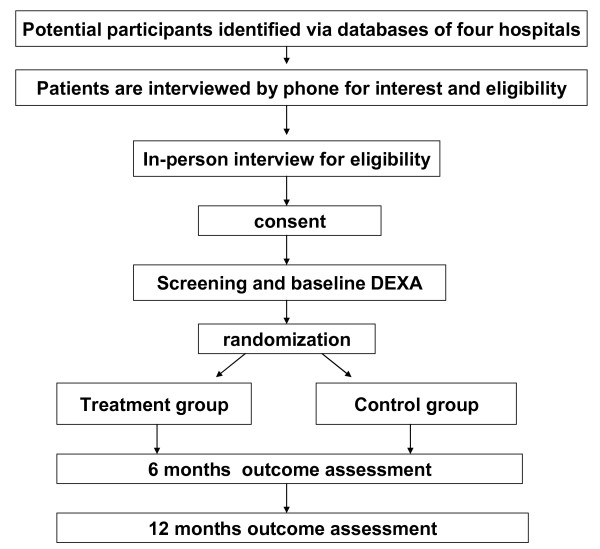
**The flow diagram of this study, including eligibility, screening, randomization and outcome assessment**.

Treatment allocation occurs when the study participant meets the inclusion criteria and signs the informed consent form. The teletherapist will then register the participant into the database, which in turn asks if they are ready to be randomized. After the teletherapist enters yes, the site-specific randomization program behind the form displays the participant's group assignment number (placebo versus Chinese medical herb). Figure 1 shows the process of patient enrollment and allocation. Study participants will be randomized in blocks of random sizes of 2 and 4 at each site. Site-specific randomization lists will be computer-generated (i.e., generated by an individualized basic visual code program) and concealed from the researchers by a senior data manager who is not involved in the study. This information will remain confidential and is not shared with the study sites, in concordance with the CONSORT guidelines. This trial uses a prospective, randomized, outcome-blinded design, in which all outcome assessments are made by a research assistant blinded to treatment allocation and uninvolved in patient consent and management.

### Outcome Measurements

The primary outcome of the study is bone mineral density at 6 and 12 months after treatment with the study medication. Secondary outcomes include pain intensity, quality of life, relative changes in bone turnover markers, neuro-endocrino-immune indices, and safety.

### Assessment of bone mineral density (BMD)

Lumbar spine BMD (L1-4) and femoral BMD will be measured at three measurement points (baseline and 6 and 12 months after the treatment), using the dual energy X-ray absorptiometry densitometer obtained from Hologic, Inc.USA. The accuracy of the measurements recorded by the dual energy X-ray absorptiometry (DXA) instruments is to be evaluated by the use of serial measurements of a local spine "phantom". The variability of DXA measurements across the different participant centres will also be assessed via utilization of the same spine "phantom" technique. Using this technique, the long-term coefficient of variation of each instrument in the study is estimated as less than 1%. These "phantom" measurements will be used to adjust for any "drift" in measurements of bone densitometry during the study.

### Assessment of bone turnover

Serum samples will be collected at baseline and 6 months after the treatment for measurement of bone turnover markers, including type I collagen N-telopeptide (NTX), serum bone-specific alkaline phosphatase (BALP), serum osteocalcin (OC), serum Procollagen type I N-terminal propeptide (PINP), carboxy-terminal telopeptide of type-I collagen (CTX), and Serum procollagen I carboxy-terminal propeptide (PICP).

### Health-related quality of life

The ECOS-16 questionnaire is developed with the aim of measuring health related quality of life (HRQoL) in postmenopausal women with osteoporosis. It is based on the combination of two disease-specific HRQoL questionnaires for women with osteoporosis: the Osteoporosis Quality of Life Questionnaire (OQLQ) [3] and the Quality of Life Questionnaire of the European Foundation for Osteoporosis (QUALEFFO) [4]. The 16 items in the new questionnaire are divided qualitatively into four dimensions. The nature of the four dimensions also suggests that they can be further combined to produce two summary scores that would include Physical Function and Pain in one Physical score and another that would include Fear of Illness and Psychosocial Function in a Mental score. These two summary scores could, in turn, be combined to provide an overall score for the questionnaire. However, although the 16 items can be classified qualitatively into four dimensions, this is a unidimensional questionnaire, according to quantitative analysis [5]. The score of each item ranges from 1 to 5. The ECOS-16 questionnaire generates a single summary score obtained from the arithmetic mean of the answered items, so the total score ranges from 1 (best HRQoL) to 5 (worst HRQoL). This questionnaire will be completed at all the measurement points (baseline and 6 and 12 months after the treatment).

### Assessment of pain

The Visual Analogue Scale (VAS) measures amount of pain, which is a pain score ranging from 0 (no pain) to 100 (worst pain ever) [6]. Operationally, the VAS score is usually a horizontal line, 100 mm in length, anchored by word descriptors at each end. The patient marks on the line the point that they feel represents their perception of their current pain. The VAS score is then determined by measuring in millimetres from the left hand end of the line to the point that the patient marks. The VAS score will be measured at all the measurement points (baseline and 6 and 12 months after the treatment).

### Assessment of adverse events

All subjects are to be questioned about adverse events (AEs) during treatment at each visit point, and all adverse events reported will be analyzed regardless of the investigators' assessments of causality. The Medical Dictionary for Regulatory Activities (MedDRA, Version 8.1J) will be used to categorize reported adverse events.

### Sample Size Considerations

We calculate the required sample size for this trial based on the following calculation: n = 2σ^2 ^× f(α,β)/(μ_1_-μ_2_)^2 ^[7]. First, we estimate that an absolute improvement of 7.8%(from μ_1 _to μ_2, _μ_1 _= 0.803, μ_2 _= 0.866) in BMD was likely the smallest clinically-relevant difference [8]. Second, we assume that standard deviation of BMD may be 0.125 (σ = 0.125) at baseline[9]. Based on these assumptions, we will require 84 patients in each group to have at least a 90% power β = 0.1 and to rule out a two-sided type I error of 5% α = 0.05. This number of patients actually provides less than 80% power, assuming an withdrawal rate of 20%. Therefore, we will recruit approximately a total of 204 patients, 102 patients in each group.

### Statistical Analysis

The data will be collected and analysed according to the intention-to-treat principle. Standard statistical techniques will be used to describe characteristics of patients in both groups. We will compare baseline characteristics in both groups and if incomparability appears, we will perform the secondary analysis, adjusting for differences. The primary outcome, BMD, will be compared between both groups using analysis of variance for repeated measures. If adjustment for possible baseline incomparability is needed, analysis of covariance will be done.

## Discussion

Osteoporosis is a major cause of morbidity and mortality in older persons. For individuals identified to be at risk for developing osteoporosis, intervention measurements are aimed at preventing the condition from getting worse, whereas in those who have already presented with low BMD or fractures, treatment is aimed at preventing further bone loss to reduce the risk of initial or subsequent fracture. Currently, there is no good evidence that these BMD differences may then translate in fracture reduction changes. However, Low bone mineral density (BMD) is a known risk factor of fracture and a strong predictor of new vertebral fracture [10,11]. A previous publication reported that after the adjustment for age and BMI, the odds of having a vertebral fracture in Southern Chinese women was 2.3(1.6-3.3) for each 1 SD reduction in spine BMD and 2.1(1.37-3.20) for femoral neck BMD [12]. A latest publication revealed that women with prevalent vertebral fractures had significantly lower BMD than those without prevalent vertebral fractures[13]. Therefore, we use BMD as the primary outcome.

Estrogens have important anticatabolic and anabolic effects on bone, and estrogen deficiency plays a central role in the development of osteoporosis [14]. Hormone replacement therapy (HRT) effectively prevents bone loss in postmenopausal women [15,16] and reduces the incidence of fractures[17,18]. However, long-term acceptance or compliance of HRT is generally poor in postmenopausal due to the potential complications, such as uterus hyperplasia and breast cancer [19,20]. A recent review suggested that many non-leguminous plants are rich in phytoestrogen, which is regarded as a prospective candidate for treatment of postmenopausal osteoporosis [21]. Among non-leguminous plants, Epimedium brevicornum maxim is a centuries-old medicinal herb. It is reputed by Traditional Chinese Medicine to have "bone strengthening" function and used for treatment of musculoskeletal disorders [22]. Epimedium-derived phytoestrogen flavonoids (EPFs) have been shown to prevent bone loss in late postmenopausal women, as indicated by the significant maintenance of BMD in the EPF treatment group compared to the placebo control group [23]. In this study, the Classic Yin and Yang Tonic Formula contains diverse phytoestrogen, such as isoflavone, lignans and coumarin. If this study demonstrates the effectiveness and safety of the Classic Yin and Yang Tonic Formula significant strides would be made towards a clinically useful therapy for reducing bone mass loss.

## List of abbreviations

BMD: (Bone mineral density); DXA: (Dual energy X-ray absorptiometry); CYYTF: (Classic Yin and Yang Tonic Formula); VAS: (Visual Analogue Scale); TCM: (Traditional Chinese Medicine); YAM: (Young adult mean); EPFs: (Epimedium-derived phytoestrogen flavonoids); NTX: (Type I collagen N-telopeptide); BALP: (Bone-specific alkaline phosphatase); OC: (Osteocalcin); PINP: (Procollagen type I N-terminal propeptide); CTX: (Carboxy-terminal telopeptide of type-I collagen); PICP: (Procollagen I carboxyterminal propeptide); OQLQ: (Osteoporosis Quality of Life Questionnaire); HRQoL: (Health related quality of life); QUALEFFO: (Quality of Life Questionnaire of the European Foundation for Osteoporosis).

## Competing interests

The authors declare that they have no competing interests.

## Authors' contributions

FY, DZT, XJC, QB, YJW, QS contributed to the design of the study. All authors contributed to the creation of the Manual of Procedures, implementation of the study protocol and acquisition of data. FY, DZT and XJC drafted the manuscript, and JDH modified the manuscript. All authors provided critical revision and approved the final manuscript.
